# N-acetyl cysteine as a potential regulator of SARS-CoV-2-induced male reproductive disruptions

**DOI:** 10.1186/s43043-022-00104-8

**Published:** 2022-06-15

**Authors:** Pallav Sengupta, Sulagna Dutta

**Affiliations:** 1 Department of Physiology, Faculty of Medicine, Bioscience and Nursing, MAHSA University, Jenjarom, Selangor Malaysia; 2 Department of Oral Biology and Biomedical Sciences, Faculty of Dentistry, MAHSA University, Jenjarom, Selangor Malaysia

**Keywords:** COVID-19, SARS-CoV-2, Male infertility, N-acetyl cysteine

## Abstract

**Background:**

The severe acute respiratory syndrome coronavirus-2 (SARS-CoV-2), causing the coronavirus disease 2019 (COVID-19), has shown its persistent pandemic strength. This viral infectivity, kinetics, and the mechanisms of its actions in human body are still not completely understood. In addition, the infectivity and COVID-19 severity reportedly differ with patient’s gender with men being more susceptible to the disease. Thus, different studies have also suggested the adverse impact of COVID-19 on male reproductive functions, mainly emphasizing on high expressions of angiotensin-converting enzyme 2 (ACE2) in the testes that allows the viral entry into the cells.

**Main body:**

The N-acetylcysteine (NAC), a potent therapeutic agent of COVID-19, may be effective in reducing the impairing impacts of this disease on male reproductive functions. NAC acts as mucolytic agent by reducing sulfide bonds in the cross-linked glycoprotein matrix in mucus owing to its free sulfhydryl group. Since NAC also breaks the viral disulfide bonds required for the host cell invasion, it may help to prevent direct SARS-CoV-2 invasion into the testicular cells as well. NAC also acts as a potent anti-inflammatory and antioxidant, directly scavenging reactive oxygen species (ROS) and regulating the redox state by maintaining the thiol pool being a precursor of cysteine (an essential substrate for glutathione synthesis). Since it is suggested that male reproductive impairment in COVID-19 patient may be caused by secondary immune responses owing to systemic inflammation and OS, the anti-inflammatory and antioxidant properties of NAC explained above may attribute in protecting the male reproduction functions from these COVID-19-mediated damages.

**Conclusion:**

This article explains the mechanisms how NAC treatment for COVID-19 may prevent the infection-mediated disruptions in male reproduction.

## Background

The coronavirus disease 2019 (COVID-19) is a unique infectious disease caused by a novel coronavirus, reported to be originated from the Wuhan city, China, in December 2019. Following its outstretch over 100 countries, the World Health Organization (WHO) asserted it as a pandemic disease on March 11 2020 [1, 2]. The disease is caused by the severe acute respiratory syndrome coronavirus-2 (SARS-CoV-2), and men are more susceptible to infection than women [3]. The genomic study of SARS-CoV-2 has explored about 79% resemblance with SARS-CoV which became epidemic in 2002 [4]. Thus, the SARS-CoV-2 may part common host cell infection contrivances with SARS-CoV. Angiotensin-converting enzyme-2 (ACE2) receptor plays a key role in the COVID-19 pathogenesis, while transmembrane protease, serine-2 (TMPRSS2) mediates priming of viral spike proteins with ACE2 [5]. The association of the host ACE2 and the spike glycoprotein of SARS-CoV-2 causes fusion of host-pathogen membrane surfaces followed by internalization of the pathogen [6].

## Main body

### Mechanism of testicular infection of SARS-CoV-2

**Fig. 1:**
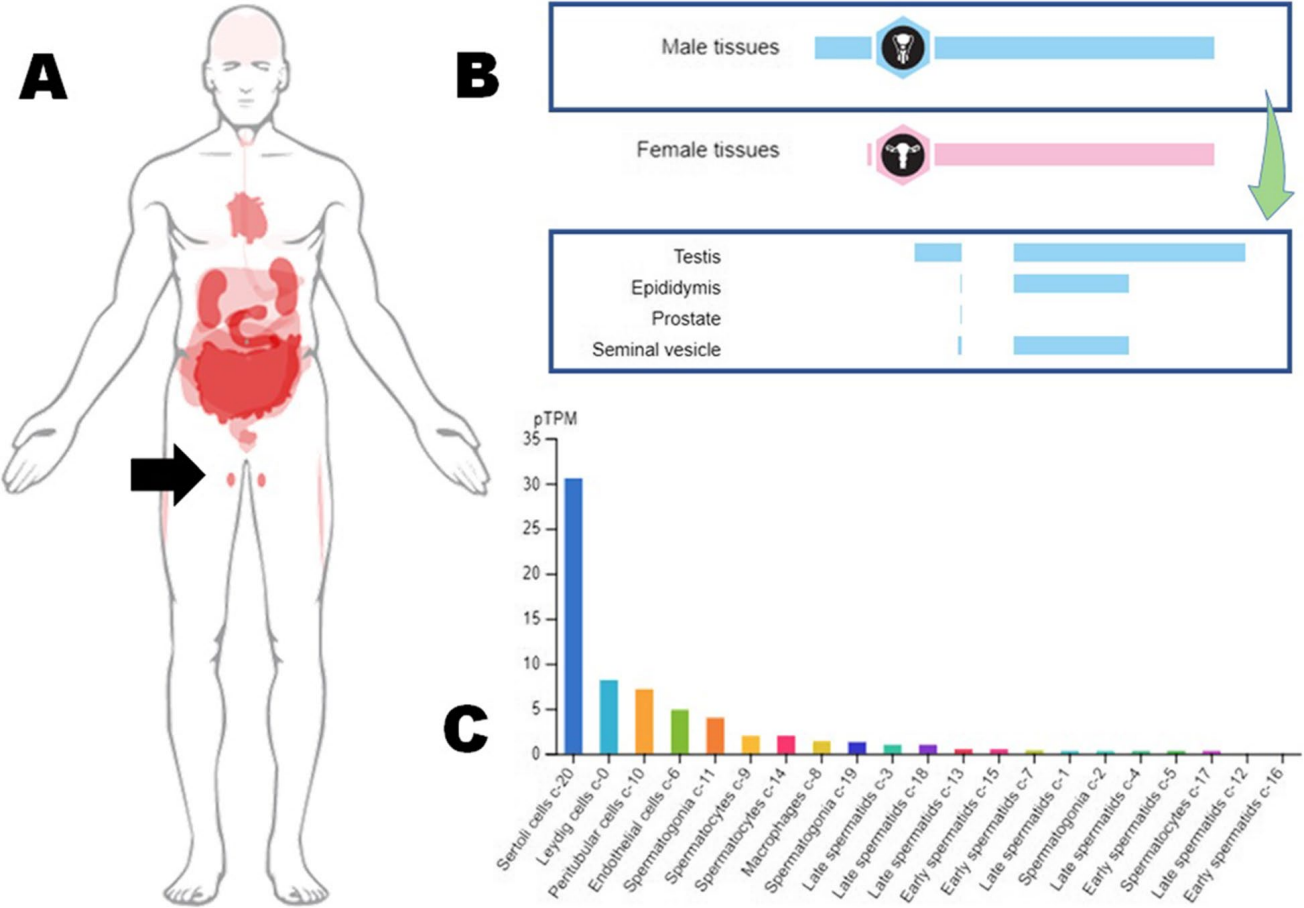
Expressions of ACE2 in different systems (**A**), male reproductive organs (**B**), and testicular cells (**C**) [source: Human Protein Atlas, 2020]

The testes have been found to have nearly the greatest levels of ACE2 mRNA and protein expression among the other bodily tissues (Fig. 1), raising the idea of a potential hazard to male fertility as a result of SARS-CoV-2 infection in these tissues [5]. Furthermore, testicular cells show much higher amounts of ACE2 than ovarian cells, whereas ovarian cells express considerably lower levels of ACE2 [7–9], which may also support higher vulnerability of male gonadal functions including steroidogenesis, towards SARS-CoV-2-mediated impairment. The activation of the androgen receptor is required for the transcription of the TMPRSS2 gene. Furthermore, the gene loci for the androgen receptor and the ACE2 enzyme are found on chromosome X [10, 11]. Polymorphisms on this chromosome, as well as endogenous androgen effects, have been linked to TMPRSS2 gene transcription and activation of the antiviral enzyme ACE2, which facilitates viral penetration in target cells [12].

The Renin-Angiotensin-Aldosterone System (RAS) is regulated by the angiotensin-converting enzyme (ACE). Through the ACE2/angiotensin (1-7)/MAS axis, ACE2 counteracts the negative effects of the ACE/RAS pathway [13]. Renin and ACE activities are required for physiological angiotensin II activation. Renin is synthesized when the juxtaglomerular apparatus (JG) of the afferent arterioles of the kidneys is activated. Renin cleaves angiotensinogen into angiotensin (Ang) I once it is released into the bloodstream. Although Ang I is not physiologically active, it is a precursor to Ang II. ACE catalyzes the conversion of Ang I to Ang II [14]. Ang II binds to angiotensin II type I and type II receptors in various tissues. Ang II activates a number of signaling pathways in cells, including serine/threonine kinase, ERK, JNK/MAPK, and PKC [15] and has been proven in studies to elevate cytokines (IL-6, TNF-α, and others), oxidative damage caused by ROS, endothelial injury caused by blocking NO generation, and vasoconstriction. The binding of spike(S) glycoprotein to ACE2 causes lesser expression of that enzyme; thus, accumulation of Ang II will take place due to the activation of ACE. This reduced ACE2 is not able to produce the vasodilator heptapeptide angiotensin 1-7 from Ang I, and this causes the initiation of lung injury. Accumulated Ang II is responsible for the overstimulation of its type-I receptor which is associated with various pathological conditions [16]. Both human ACE2 and the SARS-CoV-2 S-glycoprotein have been targeted in therapeutic strategies for the exploration of new treatment plans such as antiviral molecules and monoclonal antibodies as well as isolation of existing drug molecules which can prevent the interaction between the host and SARS-CoV-2. As for the two subunits of the S-glycoprotein, S1 facilitates the viral bonding with the host cells, whereas S2 acts as fusion protein for viral membrane [17].

The process of SARS-CoV-2 evasion depends on S-glycoprotein cleavage at S1/S2 multibasic cleavage site (MBCS), mainly with the presence of human furin, and this cleavage site plays a vital role to facilitate the entry of virus into the infected cells [18]. Furin, as termed as paired basic amino acid cleaving enzyme (PACE), has been found in the fluid from the mid-caput to the distal corpus regions of the epididymis and also in testis of various domestic mammals through immunochemistry and mass spectrometry analyses [19]. This indicated the possibility of this virus-mediated impairments in sperm production or maturation. So, the expression of *FUR* gene or inhibition of furin or obstructions and/or spoiling to form MBCS for the interaction between S1/S2 complex and furin are now the new strategies of target for the therapeutic approaches.

### N-acetylcysteine, SARS-CoV-2, and male reproduction

**Fig. 2:**
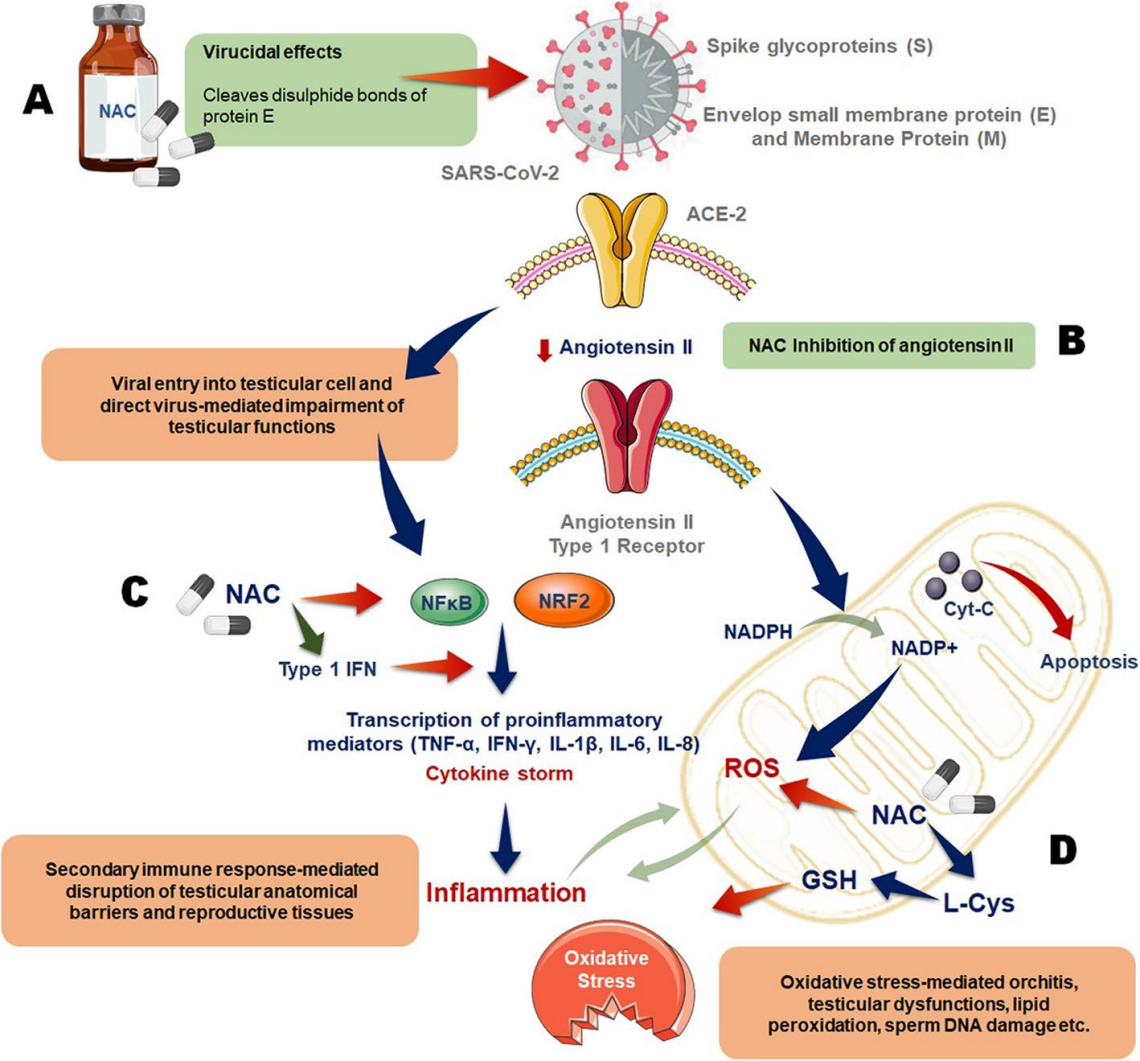
Possible mechanisms of NAC-mediated protections against male reproductive disruptions caused by SARS-CoV-2 infection. **A** Due to presence of its free sulfhydryl group, NAC reduces the sulfide bonds in the cross-linked glycoprotein matrix in mucus thus reduced mucus viscosity and showing a mucolytic property. **B** NAC may reduce the binding of angiotensin II with its type 1 receptor. NAC may block imprudent synthesis of angiotensin II which cannot be converted to angiotensin 1-7 by the activation of ACE2 thus lowering the possibility and/or severity of the infection. **C** NAC may lead to amplification of signal cascades triggered by toll-like receptor 7 and mitochondrial antiviral signal protein in restoration of SARS-CoV-2-mediated type-I interferon (IFN) production and inhibition of NFkβ (nuclear factor kappa B) mediated upregulation of pro-inflammatory genes. **D** Being a potent antioxidant, NAC may exert direct effects on some reactive oxygen species (ROS), whereas it is a precursor of cysteine (an essential substrate for glutathione synthesis) and also regulates the redox state by maintaining the thiol pool

N-acetylcysteine (NAC) has been proposed as a potential treatment, preventive and/or adjuvant against SARS-CoV-2 as it is having two major activities: first, due to presence of its free sulfhydryl group, NAC reduces the sulfide bonds in the cross-linked glycoprotein matrix in mucus thus reduced mucus viscosity and showing a mucolytic property [20, 21]. Second, being a potent antioxidant, NAC may exert direct effects on some reactive oxygen species (ROS), whereas it is a precursor of cysteine (an essential substrate for glutathione synthesis) and also regulates the redox state by maintaining the thiol pool [20]. Considering these properties, it can be hypothesized that NAC could negatively affect SARS-CoV-2 activity for the following mechanisms (Fig. 2).

#### NAC and blocking of the SARS-Cov-2 evasion route

The envelop protein or E-protein of SARS-CoV (genetically correlating to SARS-CoV-2) is ranging from 8.4 to 12 kDa in its size with the presence of 76–109 amino acids. Its primary structure is short hydrophilic amine terminus group of 7–12 amino acids, and secondary structure consists of short hydrophobic 25 amino acid transmembrane domain with carboxyl group terminus [22]. The E-protein of SARS-CoV-2 possesses a motif of triple cysteine (NH2-...L-Cys-A-Y-Cys-Cys-N...-COOH) followed by the transmembrane domain which links with another identical motif of S protein (NH2-... S-Cys-G-S-Cys-Cys-K...-COOH) [22]. Disulfide bond was recognized to be responsible for the interaction of both the motifs [22], and NAC may break those disulfide bonds and thus may prevent the possibility of host cell invasion. Thus, it may be suggested that despite the high ACE2 expressions in the testicular cells, treatment with NAC may help to prevent direct SARS-CoV-2 invasion into these cells as well. In case of sperms, studies have shown that NAC helps to maintain sperm DNA integrity by improved replacement of histones with protamine [23, 24]. Aberrant sperm protamine deposition or chromatin condensation can increase possibilities of sperm DNA damage [25], and impaired spermiogenesis (histone to protamine exchange) seems to be a major factor in male fertility [26].

Through an in vitro study, it has been observed that, in dose-dependent manner, NAC may reduce the binding of angiotensin II with its type 1 receptor [27]. NAC may block imprudent synthesis of angiotensin II which cannot be converted to angiotensin 1-7 by the activation of ACE2, thus lowering the possibility and/or severity of the infection. In one study, isosorbide dinitrate (a vasodilator) was applied to six patients for 48 h, but at 24 h, 2 g of NAC was applied followed by another dose of 5 mg/kg/h. For the first 24 h of isosorbide dinitrate application, plasma concentration of angiotensin II was increased, but NAC was applied later on; it causes the sudden fall of angiotensin II plasma concentrations from 28 ± 4 to 14 ± 2 ng/l (*p* < 0.05) [28]. This observation suggests that NAC may allow protection from the pathological effects of angiotensin II by inhibiting the ACE, which can be considered as a possible utilitarian venture in view of SARS-CoV-2 infection.

#### NAC: as a potential antioxidant

Being a potent antioxidant biomolecule, NAC may combat against the production of ROS and most importantly the cytokine storm creating the emergence of oxidative stress (OS) [29, 30]. It is supposed that the immunopathology of SARS-CoV and SARS-CoV-2 is similar and triggers an immunological response incorporating various types of pro-inflammatory cytokines like IL-1, IL-2, IL-4, TNF, and IFNs. Categorization of the IFNs follows type-I (IFN-α and β), type-II (IFN-γ), and type-III. SARS-CoV infection have been shown to suppress the type-I IFNs by impairing signal transducer and activator of transcription 1 (STAT1), antagonizing IFN. During SARS-CoV-2 infection, the above process leads to delayed IFN response by cytokine storm syndrome. NAC may lead to amplification of signal cascades triggered by toll-like receptor 7 and mitochondrial antiviral signal protein in restoration of SARS-CoV-2-mediated type-I IFN production [29]. NF-κB has been reported to cause the cytokine storm by accelerating the synthesis of several pro-inflammatory cytokines and acts as a mediator of SARS-CoV-2 pathology. This mechanism includes macrophage and neutrophil infiltrations resulting irretrievable degeneration of infected epithelium. On the other hand, an in vitro model of influenza A and influenza B has shown that administration of NAC reduced the activation of NF-κB [31] probably by restoring the thiol pools and followed by the ROS scavenging mechanism. Similarly, recent clinical study has explained that the progress of pathologic conditions may be linked with sudden fall of GSH levels and reflecting the elevated production of ROS. Therefore, cases with severe symptoms of COVID-19 infection would have been evident with increased ROS level and decreased GSH level hence the higher value of calculating ROS/GSH ratio than the cases with mild or less severe symptoms of infection [32]. Since it is suggested that male reproductive impairment in COVID-19 patient may be caused by secondary immune responses owing to systemic inflammation and OS [10], the anti-inflammatory and antioxidant properties of NAC explained above may attribute in protecting the male reproduction functions from these COVID-19-mediated damages.

Administration of 100 mg/kg continuous intravenous infusion of NAC (daily for 3 days) was also reflected a positive clinical response in a woman suffering from pneumonia, infected with influenza virus (H1N1), although oseltamivir was also applied to the patient during her treatment [33]. Besides that, administration of 600 mg of NAC (twice daily) was shown to be effective to reduce the symptoms related to influenza among the older patients (≥ 65 years) [34]. On the contrary, some studies denied the any beneficial effect of NAC administration through in vitro or in vivo model of experiments [35].

Without considering the clinical proof for the COVID-19 diagnosis, many medicines had been placed regarding the pandemic enormous health hazard; NAC is within them [36]. Administration of NAC by any of three routes (e.g., orally, intravenous, or inhaled) as an adjacent diagnosis in patients with mild symptoms of COVID-19 has been placed as less expensive clinical treatment. Recently, there are few clinical try-outs considering the probable usage of NAC for the treatment of COVID-19, as an example, efficiency and safety of nebulized heparin (NAC) in COVID-19 patients by the examination of pulmonary function improvement (HOPE) “clinical try-out is objected at governing the efficiency of nebulized NAC and Heparin in the ventilated COVID-19 patients” [37]. The objective is to create more ventilator-free days among the patients who are hospitalized with mild to severe COVID-19 symptoms. One more current study has been reported that the patients intaking NAC intravascularly 6 g/day along with other clinical diagnosis designated for COVID-19 [21, 38]. Administration of NAC orally (600 mg/day) can play a preventive measure, mainly on those carriers who are continuously been exposed to probable SARS-CoV-2 (health workers). The aforementioned application can be proven to be an important approach as several health care workers in the USA, Italy, China, Mexico, etc., had become contaminated while caring the hospitalized patients in spite of the use of personal protective equipment (PPE). This administration of NAC has been proved as beneficial not only for health care professionals but also for others those who are unable to keep them self-isolated. If the effectiveness of NAC administration is proved in a long run, then it can form a plateau in the exponential contagion curve among many countries, only after several clinical trials. The confirmation of probable use of NAC as an element in preventing the disease caused by SARS-CoV-2 needs basic laboratory and clinical studies. The previously mentioned will need to be one of the countless efforts to find out the auxiliary treatment which can be either novel or not objected at pointing the recent COVID-19 outbreak along with minimizing the contamination process from one person to other.

## Conclusion

NAC is a potent agent in the treatment of COVID-19. Because of its free sulfhydryl group, NAC functions as a mucolytic agent by decreasing sulfide bonds in the cross-linked glycoprotein matrix of mucus. NAC may also inhibit direct SARS-CoV-2 invasion into testicular cells by breaking the viral disulfide bonds essential for host cell invasion. Since secondary immune responses resulting from systemic inflammation and OS are suggested to cause male reproductive impairment in COVID-19 patients, the anti-inflammatory and antioxidant properties of NAC may contribute to protecting male reproduction functions from COVID-19-induced damage. The article provides an idea regarding the possible processes by which NAC treatment for COVID-19 may prevent infection-mediated abnormalities in male reproduction, and thus, it encourages in-depth studies in this field so that the clinical potencies of NAC as male reproductive ameliorator in SARS-CoV-2 infections are completely revealed.

## Data Availability

Not applicable.
